# The Influence of Formaldehyde Fixation Media on the Raman Spectroscopic Analysis of Oral Squamous Cell Carcinoma

**DOI:** 10.1002/jbio.70326

**Published:** 2026-07-26

**Authors:** Levi Matthies, Jasper F. Tegtmeyer, Caroline Pessler, Medhanie T. Gebrekidan, Katharina H. Besch, Ralf Smeets, Andreas S. Braeuer, Martin Gosau, Christian Knipfer

**Affiliations:** ^1^ Department of Oral and Maxillofacial Surgery University Medical Center Hamburg‐Eppendorf Hamburg Germany; ^2^ Technische Universität Bergakademie Freiberg (TUBAF), Institute of Thermal‐, Environmental‐ and Resources Process Engineering (ITUN) Freiberg Germany; ^3^ Department of Oral and Maxillofacial Surgery, Division of “Regenerative Orofacial Medicine” University Medical Center Hamburg‐Eppendorf Hamburg Germany

**Keywords:** biophotonics, formalin fixation, oral squamous cell carcinoma, Raman difference spectroscopy, shifted excitation Raman difference spectroscopy

## Abstract

Raman Difference spectroscopy (RDS) presents a promising, non‐invasive approach for facilitating rapid diagnosis of oral squamous cell carcinoma (OSCC). Many ex vivo studies rely on formalin‐embedded tissue specimens. Spectroscopic changes and classification of formalin‐fixed OSCC tissues were investigated. In an analysis of 47 specimens (*n =* 30 OSCC, *n* = 17 physiological mucosa), RDS was employed at 180 distinct measurement loci after storage in saline, and again at 125 measurement loci after storage in formalin. For classification, Principal Component Analysis (PCA) and Linear Discriminant Analysis (LDA) were utilized. In distinguishing OSCC from physiological mucosa, LDA revealed a higher sensitivity (97.6% for NaCl‐stored vs. 90.9% for formalin‐fixed tissues), specificity (93.0% vs. 78.6%), and overall classification accuracy (95.2% vs. 85.4%) for specimens stored in saline. While formalin fixation is valuable for tissue preservation in histopathology, Raman spectra of OSCC tissues were substantially altered, with implications for validation of innovative optical techniques such as Raman Spectroscopy.

## Graphical abstract

Formalin fixation substantially altered the Raman spectra of OSCC tissues. In distinguishing OSCC from physiological mucosa, LDA revealed a higher sensitivity (97.6% for NaCl‐stored vs. 90.9% for formalin‐fixed tissues), specificity (93.0% vs. 78.6%), and overall classification accuracy (95.2% vs. 85.4%) for specimens stored in saline.

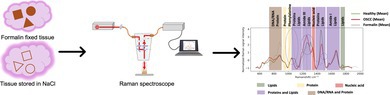

## 1 Introduction

Oral cancer is among the most common malignancies worldwide, with a global incidence estimated by the WHO International Agency for Research on Cancer at 389 846 new cases and 188 438 deaths in 2020 [1, 2, 3]. Herein, oral squamous cell carcinoma (OSCC) constitutes 95% of all malignant tumors of the oral cavity [4, 5]. Current guidelines for the diagnosis of oral mucosal lesions suggest that any abnormalities should be closely monitored initially and treated with conservative measures. In case of high suspicion or if a mucosal lesion persists, surgical biopsy and histopathological examination should be performed as the standard of care [6, 7, 8].

However, sample collection and analysis are time‐consuming with a delay in time‐to‐diagnosis and are prone to error due to inter‐ and intra‐individual variation, both in surgery and pathology [9, 10, 11, 12]. These limitations can be attributed to the pathologist's expertise and experience [9], tissue shrinkage after resection and fixation especially in the examination of tumor margins [13], and the difficulty of detecting minimal residual tumor cells [14]. Additionally, the quality of samples can be compromised by surgical instruments and thermal damage [13, 15]. Therefore, the use of an optical imaging method that is non‐invasive, real‐time and sample‐free could overcome these limitations for in vivo approaches. There is currently no objective instrumental method for rapid, real‐time diagnosis of the abnormal mucosal lesion or surgical margins in daily clinical practice.

To address this, optical technologies for the early diagnosis of OSCC are being investigated. Among these, Raman spectroscopy (RS) provides specific information about the molecular composition and structure of biological tissue. RS is based on the inelastic interaction of light with matter, known as the Raman effect [6]. Thus, information about molecular transitions can be obtained spectroscopically. Since the transitions are molecule‐specific, the resulting Raman spectrum provides a “chemical fingerprint” of the biological tissue being analyzed [16].

Raman spectroscopy in biomedical optics thus offers the possibility to acquire high‐quality spectral information at the molecular level. This data can be obtained with little or no sample preparation, label‐free and without time delay, while working non‐invasively, which makes it a perfect tool for chairside diagnostics in many medical fields with a great potential for ex vivo and in vivo applications [17, 18, 19, 20, 21, 22, 23, 24, 25, 26, 27].

In the specific domain of oral cancer research, our group has shown the considerable potential of Raman spectroscopy in a series of studies. We have demonstrated the ability of Raman spectroscopy to reliably detect OSCC and differentiate between OSCC and physiological mucosa [28, 29]. Furthermore, the group has worked to improve the acquisition and evaluation of Raman spectra through the implementation of technical approaches and artificial intelligence methods [20, 30, 31, 32].

Herein, the widely established preservation of tissue samples in formalin for ex vivo measurements plays a pivotal role regarding the application of Raman spectroscopic analysis. Most Raman‐based studies have been performed on formalin‐fixed tissues to obtain preliminary results or data for basic preclinical studies [24, 33, 34, 35, 36, 37]. While formalin fixation of biological tissues can be used to preserve the state of cells and tissues, the sample preparation is associated with direct spectroscopic interferences due to the formalin itself [38, 39]. It was shown that formalin fixation leads to spectral alterations based on changes in molecular composition [40, 41]. Formalin fixation causes the deactivation of tissue proteases which then stops the cells' degeneration and furthermore influences the protein of the tissue. Formalin‐protein interactions are influenced by cross‐linking of formalin with defined amino acids [38, 42, 43, 44].

With the increasing amount of spectral data on biological tissues, it is of great interest to avoid misinterpretation of spectra by determining the exact influence of tissue processing on the collected spectral data. Therefore, the aims of the present study are to firstly evaluate the spectroscopic changes that occur due to formalin fixation in OSCC tissue. The second aim is to identify further potential limitations of formalin‐fixed tissue in differentiating between OSCC and physiological mucosa, by directly comparing the results to those obtained from native tissue samples. To achieve this, the measurements were performed on the same sample that was initially stored in NaCl and subsequently in formalin.

## 2 Materials and Methods

### 2.1 Raman Difference Spectroscopy

The measurements were performed with a self‐developed hardware and software Shifted Excitation Raman Difference Spectroscopy (SERDS) setup, which has been described comprehensively in previous work of our research group [20, 28, 30, 31, 32]. The schematic setup of the self‐developed Raman sensor for the investigation of oral cancer tissues is shown in Matthies et al. in 2021 [29]. Briefly, a diode laser (DLpro, Toptica photonics, Munich, Germany) with a variable laser wavelength between 770 and 810 nm was used as the excitation light source. The excitation wavelengths of 779 and 780 nm were chosen as the parameters for the difference spectroscopy measurements.

### 2.2 Tissue Specimens and Spectral Data Acquisition

The present study is based on the analysis of a total of 47 tissue samples, comprising 30 samples of oral squamous cell carcinoma (OSCC) and 17 samples of physiological oral mucosa from 29 patients. After obtaining patients' informed consent, the samples were taken intraoperatively from the central aspect of histopathologically confirmed OSCC resections and their corresponding surgical safety margins of > 5 mm (i.e., healthy, physiological mucosa). The samples were stored temporarily in their native state (i.e., isotonic, buffered 0.9% NaCl solution) at 4 C for a maximum period of 6 h until the measurement in their native state was conducted. Following the collection of spectroscopic data from the native tissue samples, each sample was fixed by immersion in 5 mL of 3.6% neutral buffered formalin (Fischar, Saarbrücken, Germany) and stored at room temperature (22°C) for a minimum of 48 h prior to a second measurement of the same sample.

The study comprised an investigation and differentiation of the spectral characteristics of physiological tissue and OSCC to define differentiation features on native tissue samples. This part of the study included all 47 samples of the 29 patients (*n* = 30 OSCC; *n* = 17 physiological mucosa). Furthermore, the specific impact of formalin fixation on Raman spectra and histopathologic discrimination between physiological mucosa and OSCC was investigated. To this end, the Raman spectrum of formalin was compared with the spectra of the tissue samples after storage in formalin. Due to fixation‐induced tissue shrinkage, fewer independent measurement loci were available in formalin‐fixed specimens while maintaining the predefined minimum spacing criterion (evenly distributed, with a minimum distance of 2 mm in between and from the peripheral tissue margins).

After spectral data acquisition, comprehensive spectral data pre‐processing to reduce background and noise was employed as described in detail previously and in brief as follows: [28, 29, 31, 34]. The two mean raw spectra, one for each excitation wavelength (779 and 780 nm, focal spot diameter of approximately 200 μm and axial resolution of approximately 5 μm), were first *z*‐score normalized. Then a Raman‐difference spectrum was calculated. As the fluorescence background is expected to not be influenced by the excitation wavelength, most of the fluorescence background was eliminated by the subtraction. However, due to photo‐bleaching, the resulting difference spectrum still contained fluorescence residuals. The residual fluorescence, already vastly reduced, was eliminated further using mathematical approaches. First, the center of the difference spectrum was identified using an asymmetric least squares fit and subtracted from the difference spectrum. In this context, denoting the center fitting of the difference spectrum from the SERDS technique, which is utilized for the purpose of reducing any residual fluorescence that remains following subtraction. The first step in the process is the normalization of the spectra at the two SERDS wavelengths. After the subtraction process, a center fitting technique was employed to eliminate any residual fluorescence. Then a spectrum was reconstructed from the center‐corrected difference spectrum using a mathematical recurrence relation. The obtained reconstructed spectrum still contained a weak fluorescence background which was eliminated further by applying a baseline correction based on piecewise asymmetric least squares fitting [45]. Finally, a reconstructed Raman spectrum was obtained.

The spectra were correlated with the classified tissue entities after histopathological confirmation. This approach enabled an intra‐individual comparison of the differentiability between OSCC and physiological tissue before and after formalin fixation using the same tissue samples, thereby ensuring a highly valid outcome.

### 2.3 Statistical Analysis

Data analysis included data processing using principal component analysis (PCA) to identify the most significant spectral patterns of the tissue samples, and classification of the biological tissues examined based on linear discriminant analysis (LDA). For this purpose, two separate LDA‐based classification models were built [46]. Each model was tested by a fivefold cross‐validation, in which the spectral data set was divided into training and test data sets in an iterative loop. Per cross‐validation iteration, approximately 80% of the dataset was used to train the model and approximately 20% of the dataset was used to test its predictive ability. Furthermore, a split internal cross‐validation was implemented to calculate the optimal number of main components and hyperparameters of the classifier based on a sequential model‐based optimization. The number of principal components used in the LDA was determined by internal cross‐validation to prevent overfitting. Each classifier was assigned an equal distribution of both classes for training, using methods for resolving data imbalances. In this way, any unintentional bias of the classifiers towards classes with minority datasets was avoided as far as possible [47].

## 3 Results

A total of 11 900 raw spectra were acquired from 30 OSCC tissue samples, including 119 distinct measurement loci, and these were subsequently processed into 238 mean reconstructed Raman spectra. For physiological mucosa, a total of 6100 raw spectra were acquired from 17 tissue samples, derived from 61 measurement loci, from which 122 mean reconstructed Raman spectra were subsequently analyzed (see Table 1).

**TABLE 1:** Overview of patients, specimens and measurement loci.

	OSCC	Physiological mucosa
Tissue samples	30	17
Measurement loci	119	61
Raw spectra	11 900	6100
Mean spectra	238	122
Total samples	47	
Total patients	29	

**FIGURE 1:**
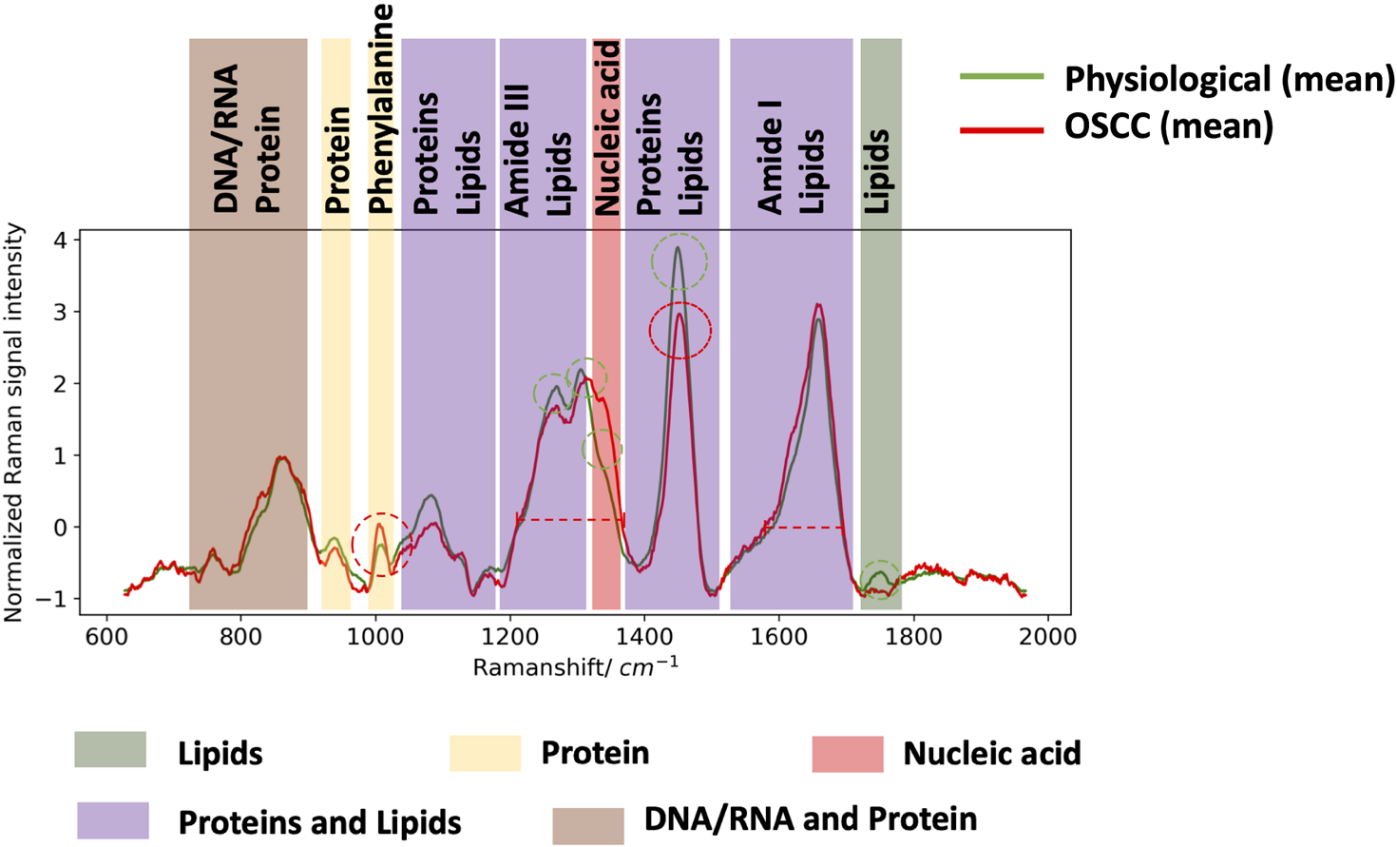
Reconstructed mean Raman spectra of physiological oral mucosa (green line) and OSCC tissue (red line) stored in NaCl with peak positions assigned to their respective molecular origin. Lipids (light green), proteins (light yellow), both proteins and lipids.

To establish the differentiation features of the various tissues, the reconstructed Raman spectrum for OSCC tissue and for physiological oral mucosa stored in sodium chloride solution were averaged over all 30 OSCC samples and 17 physiological mucosa samples, respectively. Figure 1 illustrates these averaged reconstructed Raman spectra, in which the corresponding spectral Raman peaks are assigned. Table 2 provides a summary of the spectral Raman features and their respective molecular origins, as referenced in the literature to compare with our results [48, 49].

**TABLE 2:** Assignment of the spectral features in the mean Raman spectrum to the molecular vibrations and origin [48, 49].

Physiological mucosa	OSCC	Molecular origin	Main assignment
1003	1002	Phenylalanine	Protein
	1340	Nucleic acid	
1448		CH_2_ bend/deformation	Lipid
	1450	CH_2_ bend	Protein
1656		C=C	Lipid
	1658	Amid‐I (α‐helix)	Protein
1750		C=O (Lipid in healthy mucosa)	Lipid

**FIGURE 2:**
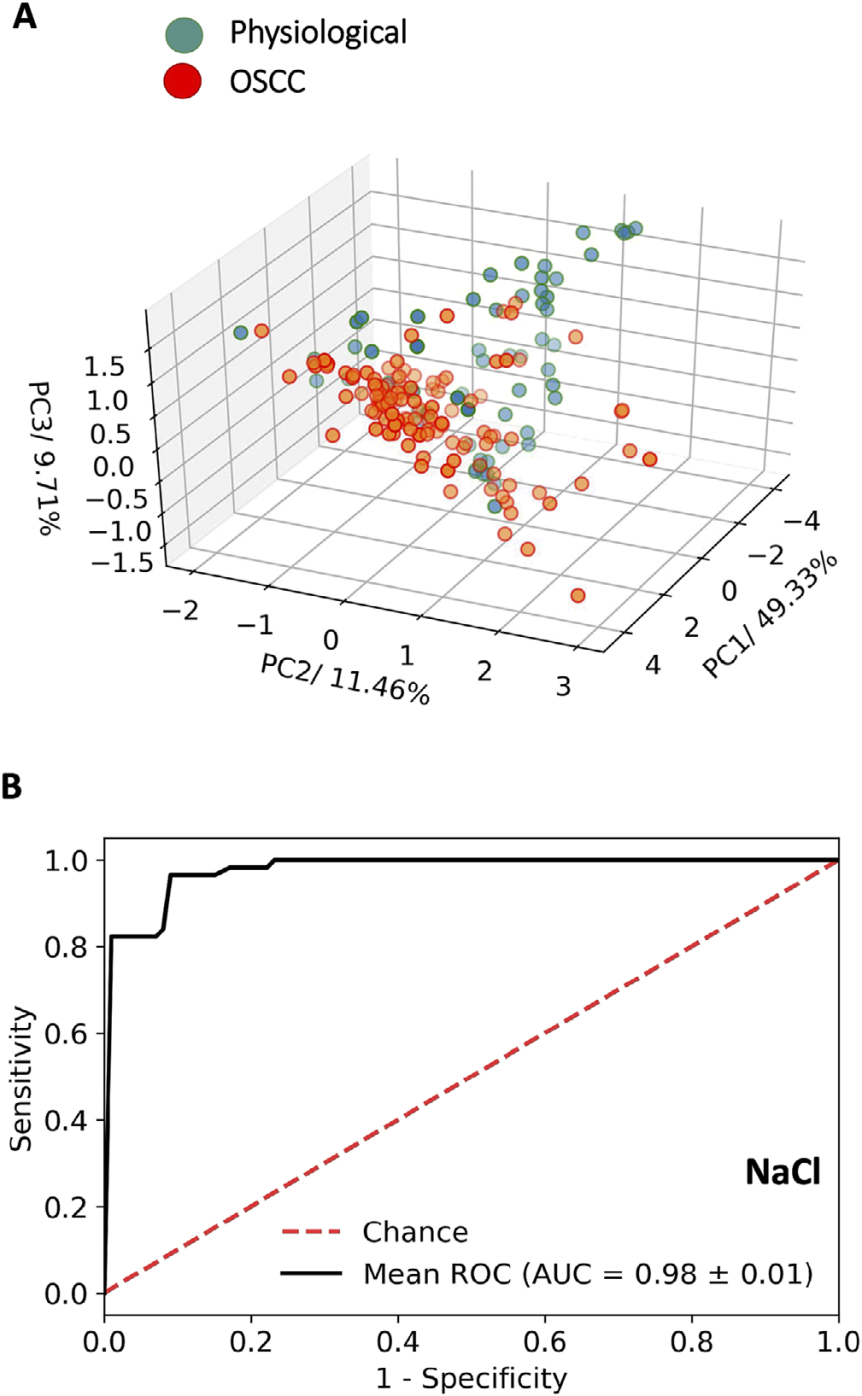
(A) Results of principal component analysis of generated Raman spectra of OSCC lesions (*n* = 30; red dots) and physiological oral mucosa (*n* = 17; green dots) stored in NaCl from a total of 47 patients. (B) Receiver Operating Characteristic (ROC), to evaluate the performance of the PCA‐LDA classifiers between healthy oral mucosa versus OSCC tissue (black curve) stored in NaCl.

PCA and LDA were employed to assess the differentiability of physiological oral mucosa and OSCC tissue stored in NaCl, with the results presented in Figure 2A as a principal component plot. As can be seen from the PCA cluster, these show clear differences in their visual spatial arrangement with minimal overlap, confirming previous findings of our group in this dataset. In this data set, the first principal component contains 49.33% of the spectral variation of the two tissue types, while the second principal component comprises 11.46% and the third 9.71%. The performance of the classifiers employed is illustrated as a Receiver Operating Characteristic (ROC) in Figure 2B. The classification of physiological oral mucosa versus OSCC tissue stored in NaCl was achieved with a five‐fold cross‐validation accuracy of 93.9%, a sensitivity of 97.5%, and a specificity of 96.9%. The area under the curve (AUC) is determined to be 0.98, with only 11 of the 180 measurement loci being assigned to a misclassification, corresponding to a classification error of 6.1%.

**FIGURE 3:**
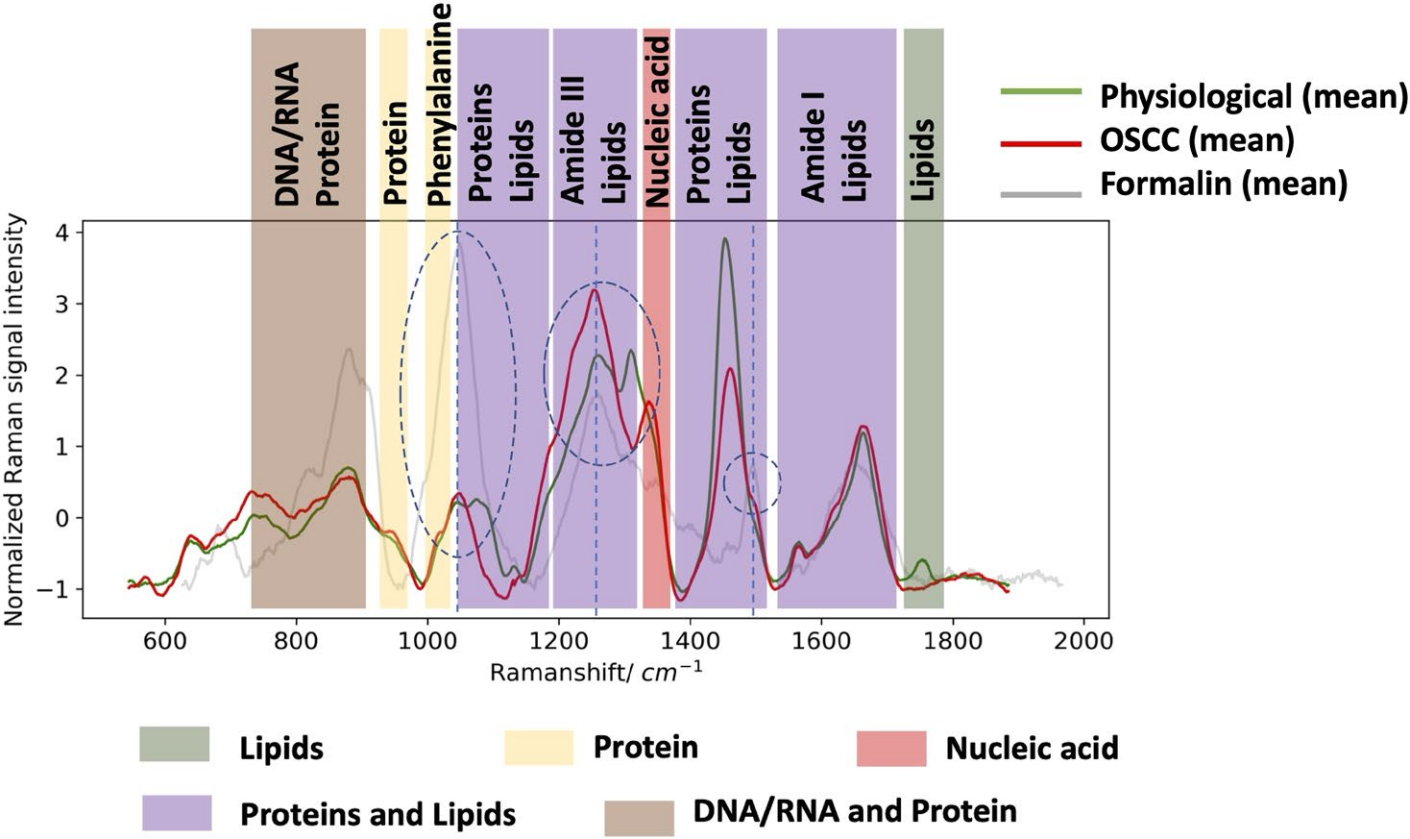
Reconstructed mean Raman spectra of formalin‐fixed physiological oral mucosa (green line) and formalin‐fixed OSCC tissue (red line) with assignment of peak positions to their respective molecular origin. The grey line corresponds to the reconstructed mean Raman spectrum of formalin. Dotted lines and circles are used to highlight the effect of formalin.

Afterwards, measurements were performed on the same samples after fixation in formalin to investigate its influence on the Raman spectrum. Figure 3 illustrates the averaged Raman spectra for OSCC tissue and physiological oral mucosa stored in formalin and the spectrum of formalin. The peak of phenylalanine was observed to shift from 1004 to 1039 cm^−1^, and a change in the CH_2_ band was detected. This was found to be broadened and co‐localizes with the peak of formalin at 1448 cm^−1^ in the so‐called fingerprint region from 1200 to 1800 cm^−1^ [29].

As another part of the study, an analysis was carried out to determine the spectral difference between samples stored in NaCl and afterwards in formalin and its impact in distinction between OSCC and physiological mucosa. Nine patients with corresponding samples were selected for this purpose to reduce interindividual variability as a potential confounder. A total of 4100 (OSCC) and 4300 (physiological mucosa) raw spectra were included for physiological oral mucosa and OSCC tissue stored in NaCl, respectively. These were acquired at 41 and 43 measurement loci, from which 82 and 86 mean reconstructed Raman spectra could be extracted and included in the evaluation. For tissue samples that were fixed in formalin, a total of 2200 and 1900 raw spectra were acquired for physiological oral mucosa and OSCC tissue, respectively. Based on 22 and 19 measurement loci, from which 44 and 38 mean reconstructed Raman spectra were extracted. Table 3 provides a detailed list of the distributions described.

**TABLE 3:** Number of patients, distribution of tissue samples, collected raw and mean spectra, and measurement loci regarding discrimination between healthy oral mucosa and OSCC tissue (in NaCl and formalin).

	NaCl		Formalin	
	OSCC	Physiological mucosa	OSCC	Physiological mucosa
Tissue samples	9	9	9	9
Measurement loci	41	43	22	19
Raw spectra	4100	4300	2200	1900
Mean spectra	82	86	44	38
Total tissue samples	18			
Total patients	9			

**FIGURE 4:**
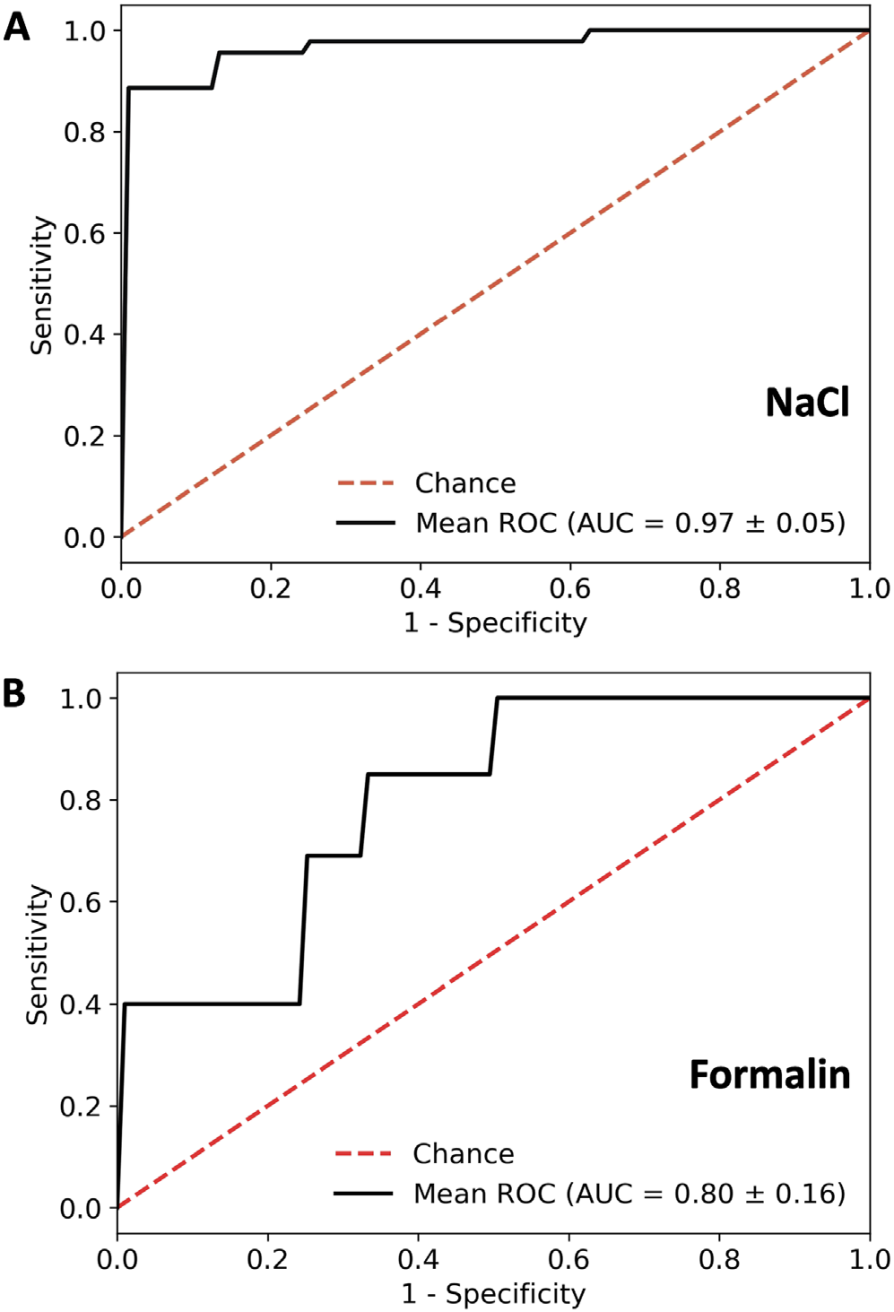
Receiver operating characteristic curve (ROC) to evaluate the performance of the PCA‐LDA classifiers. (A) NaCl stored healthy oral mucosa vs. OSCC tissue (black curve), resulting in an AUC of 0.972. (B) In comparison, formalin‐fixed healthy oral mucosa was discriminated against OSCC tissue (black curve) with an AUC of 0.799.

LDA was used to investigate the classification of physiological tissue and OSCC tissue under the influence of different storage media. Two LDA classification models were created to discriminate between the data sets of physiological oral mucosa and OSCC tissue stored in NaCl and formalin. Figure 4 shows the corresponding receiver operating characteristic (ROC) curves for the different storage media, which visualize the performance of the classifiers used and thus show their performance in relation to the different storage media. For the classification of physiological oral mucosa stored in NaCl versus OSCC tissue, a fivefold cross‐validation accuracy of 95.2% was achieved with a sensitivity of 97.6% and a specificity of 93.0%. The area under the curve (AUC) was determined to be 0.972, with 4 out of 84 loci being misclassified, giving a classification error of 4.8% (see Figure 4A).

For the classification of formalin‐fixed physiological mucosa versus OSCC tissue, a fivefold cross‐validation accuracy of 85.4% was achieved with a sensitivity of 90.9% and a specificity of 78.9%. The area under the curve (AUC) was determined to be 0.799, with 6 out of 41 loci misclassified, resulting in a classification error of 14.6% (see Figure 4B).

A further objective of this study was to examine the potential impact of disparate tissue sample collection sites within the oral cavity on the resulting analysis. To this end, tissue samples from a cohort of patients (*n* = 13), stored in NaCl, were analyzed at 12 measurement loci each, with four different sampling areas (alveolar ridge, floor of the mouth, tongue, cheek) being considered. A total of 1200 raw spectra were acquired for each sampling area and included in the analysis as 24 mean reconstructed Raman spectra. Subsequently, PCA was employed to ascertain discrepancies between the OSCC tissue samples of disparate anatomical sites. The results are presented in the form of a two‐dimensional diagram (see Figure S1 and Table S1), arranged according to the first two principal components (PC1 and PC2). In this data set, the first principal component accounts for 33.50% of the spectral variation observed across the four anatomical regions, while the second principal component accounts for 18.56%. As shown in the principal component plot, the data clustering demonstrates no notable distinction in terms of topographical origin, exhibiting a substantial spatial overlap.

## 4 Discussion

Optical methods such as Raman spectroscopy are powerful techniques that can non‐invasively differentiate between physiological and tumor tissues based on their spectral signatures [16, 50, 51]. To explore the quality of tissue analysis using Raman spectroscopy, our group has recently demonstrated approaches for the investigation of dermal neurofibroma and antiresorptive drug‐related osteonecrosis of the jaw in NaCl‐stored specimens [20, 52]. Several studies have shown the potential of Raman spectroscopy for early cancer detection, and the literature indicates promising results for head and neck cancer diagnosis [16, 20, 21, 23, 24, 29, 30, 52, 53, 54]. In OSCC, a high degree of accuracy has been reported in the differentiation of malignant lesions from tissue samples of normal mucosa [29, 55]. Our results demonstrate the differentiation of OSCC from physiological mucosa in a highly valid manner and the negative impact of formalin fixation of tissue samples on this issue. In ex vivo approaches to tissue analysis using Raman spectroscopy, there is often no distinction between fresh and formalin‐fixed tissue. Few studies have investigated the effect of formalin on Raman spectroscopy acquisition, particularly in oncologic research.

One aspect is the potential for spectral changes following formalin fixation. It has been shown to cause a significant decrease in the intensity of Raman spectra in tissue samples [38], which may result in the loss of important diagnostic markers or changes in spectral features that are critical for accurate tissue differentiation. If the formalin spectrum dominates the samples, the tissue features will be less pronounced, making them more difficult to detect. Formalin renders OSCC and physiological mucosa virtually identical because both spectra are now equally dominated by formalin. The decrease in spectral quality in formalin‐fixed tissue samples has been attributed to several factors, including the leaching of chemical species during fixation [56], the formation of cross‐links or changes in protein conformation [41] and direct spectral contamination from the formalin itself [38, 39].

In this study, we were able to show—for the first time in the field of oral cancer—that there is a clear influence of formalin in the measured Raman spectra. In particular, the phenylalanine peak was shifted from 1004 to 1039 cm^−1^ and we observed a change in the CH_2_ band, which was broadened and co‐localizes with the peak of formalin at 1448 cm^−1^ in the so‐called fingerprint region from 1200 to 1800 cm^−1^ [29]. Both spectral changes lead to obstructed tissue classification, as they affect spectral features that are relevant for tissue differentiation. These important spectral features have been described in previous studies by our group [28, 29].

In discriminating between physiological oral mucosa and OSCC tissue, we demonstrated higher sensitivity (97.6%), specificity (93.0%), and overall accuracy (95.2%) for tissue stored in NaCl. In comparison, the resulting classification accuracy for formalin‐stained tissue was 85.4%, with a sensitivity of 90.9% and a specificity of 78.9%. The classification error for formalin‐stained tissue (14.6%) was considerably higher than for NaCl stored tissue (4.8%), in a higher chance of misclassification after formalin fixation, in the light of oncologic diagnosis. By comparing the mean reconstructed Raman spectra, it is possible to differentiate specific spectral characteristics between physiological oral mucosal tissue and OSCC tissue. As our group has already demonstrated in previous studies a pivotal differentiating factor is the peak observed at approximately 1750 cm^−1^. This is due to the vibration of the C=O lipid bond and is exclusive to the Raman spectrum of samples from physiological oral mucosa. Another distinctive feature between OSCC and physiological oral tissue is the presence of a peak or band in the amide‐range at approximately 1656 cm^−1^. Furthermore, the spectral range around 1448 cm^−1^ can also be identified as a valid distinguishing feature. Conversely, in OSCC tissue, both the prominent spectral feature of phenylalanine around 1003 cm^−1^ and the prominent peak of the nucleic acid (1340 cm^−1^) prove to be distinguishing criteria of the Raman spectra [28, 29].

Other studies support our findings regarding the influence of formalin, describing spectral contamination in formalin‐fixed tissue as well as changes in peak interferences [38, 40]. Analysis of formalin‐fixed tissue may not reveal the full biochemical characteristics of tissue samples, making it difficult to extrapolate results to in vivo approaches [56]. Mirizzi et al. also described spectral changes due to formalin compared to cryofixation when analyzing neuronal tissue with Raman spectroscopy, but also a good signal‐to‐noise ratio that still allows distinguishing between tumor and physiological tissue [40].

A review of the accuracy of fiber‐optic Raman spectroscopy for the detection of head and neck cancers published in 2023 supports Raman spectroscopy as a powerful tool for head and neck cancer diagnostics [53]. By comparing 10 studies with in vivo approaches of Raman spectroscopy, a pooled sensitivity and specificity of 0.88 and 0.94 were observed [20, 28, 29, 30, 52]. The overall diagnostic accuracy was 0.96, which implements a high potential of Raman spectroscopy as a diagnostic tool even though there was a high heterogeneity of the compared studies [53]. More studies are needed in the future using standardized settings with larger, multicenter cohorts.

One confounding factor in our study is that we used fewer measurement loci for spectral data acquisition of formalin‐fixed samples. First, formalin fixation causes measurable tissue shrinkage in oral mucosal specimens, which is particularly relevant for histopathological assessments of surgical margins. Studies of head and neck cancer in general, and oral squamous cell carcinomas in particular, have shown that formalin‐induced dehydration and protein cross‐linking can reduce tissue dimensions by 4%–28% [57, 58, 59]. Furthermore, mucosal tissues are particularly susceptible to shrinkage due to fixation because of their high water content and loose connective tissue architecture [58, 60]. These dimensional alterations may result in fewer measurement loci due to the true sample size after fixation. Nevertheless, reliable spectral quality was demonstrated, even with fewer measurement loci, and relevant differences between the two storage media were revealed.

To address potential confounding factors affecting OSCC diagnosis by RS, anatomical subsites were analyzed. It is evident that the oral cavity is anatomically heterogenous, exhibiting subtle variations attributed to the presence or absence of thick, stratified, squamous, non‐keratinizing mucosa in specific locations. Such variations can be discriminated with Raman spectroscopy [61]. Sahu et al. reported significant spectral differences between the buccal mucosa, lip, and tongue, particularly in relation to lipid and protein content, which were especially pronounced in the affected and healthy contralateral conditions [62]. However, no significant differences were observed in the spectra from premalignant and malignant tissue for all subsites. The distinction between physiological mucosa and malignant lesions was marginally more precise in the diagnostic algorithm for the subsites. Remarkably, the classification algorithm encompassing all subsites demonstrated a sensitivity of 98% and a specificity of 100%. In contrast, Krishna et al. stated that subsite‐anatomical differences did interfere with healthy and pathological distinction [63]. In our study, no notable distinction was found in terms of topographical origin, with a substantial spatial overlap. However, this may also be due to differences in experimental setup. A further potential explanation pertains to the occurrence of malignant transformation and de‐differentiation of cell clusters within neoplastic lesions, thereby obstructing their accurate categorization as mucosal regions of the oral cavity [64]. Nevertheless, we acknowledge that the anatomical location may be a potential confounding factor, and that further investigation is required. Despite convincing results, our study is limited by the small size of the available cohort to assess the impact of formalin fixation on differentiating between OSCC and physiological mucosa. We used all native samples to evaluate the performance of the PCA‐LDA classifiers with a larger sample size. Spectra from all formalin‐fixed samples were included to characterize formalin‐related spectral features and the associated alterations within diagnostically relevant regions. To conduct a comparative analysis between formalin‐fixed and native tissue, the cohort was reduced to corresponding samples from the same patients to ensure direct comparability when distinguishing between physiological mucosa and OSCC. This matched‐sample design is a methodological strength because it minimizes interindividual biological variability as a potential confounding factor and allows us to attribute the observed spectral differences specifically to the effects of formalin fixation. Consequently, further studies including larger patient cohorts and subgroup analyses are necessary to validate these findings.

## 5 Conclusion

Although formalin fixation is advantageous for tissue preservation and histopathological evaluation, the established diagnostic gold standard and current reference, its effect on optical techniques such as Raman spectroscopic analysis should be recognized. The changes in spectral features may limit the reproducibility and accuracy of Raman spectroscopic findings, particularly with regards to validation for potential in vivo application and oral cancer diagnosis. While mathematical approaches may help to eliminate spectral interference from formalin fixation, our study shows that a decrease in spectral quality in formalin‐fixed tissue samples remains. This technique can be useful in the analysis of a larger collective in the future. We hope to contribute to improving the accuracy of optical OSCC evaluation and, ultimately, enhance both diagnosis and treatment of patients affected by this disease. Further studies are needed in head and neck oncology and on the implementation of Raman spectroscopy as a future chairside tool.

## Author Contributions

Investigation: L.M. and J.F.T. Formal analysis, methodology and software: M.T.G. and A.S.B. Resources and project administration: M.G. and R.S. Funding acquisition: C.K. and A.S.B. Writing – original draft preparation: C.P. and K.H.B. Writing – review and editing: A.S.B., C.K., and L.M. All authors have read and approved the final manuscript.

## Funding

This study was supported by the Wilhelm Sander Foundation (grants 2017.111.1 and 2017.111.2).

## Ethics Statement

The study protocol is in accordance with the Declaration of Helsinki and has been reviewed and approved by the local Ethics Committee of the universities of Erlangen‐Nürnberg (AZ 243_12 B) and Hamburg (AZ MC‐309/17 and AZ PV7012).

## Consent

Informed consent was obtained from individuals prior to participation.

## Conflicts of Interest

The authors declare no conflicts of interest.

## Supporting information


**Figure S1:** Results of Principal Component Analysis (PCA) of Raman spectra from OSCC tissues of different anatomical origin.
**Table S1:** Number of tissue samples from four areas of the oral cavity, measurement loci, number of raw and mean spectra and number of patients we collected the samples from with regard to examine the potential impact of disparate tissue sample collection sites within the oral cavity.

## Data Availability

The data that support the findings of this study are available on request from the corresponding author. The data are not publicly available due to privacy or ethical restrictions.
