# Supplementary material for: The Influence of Formaldehyde Fixation Media on the Raman Spectroscopic Analysis of Oral Squamous Cell Carcinoma
Source: J Biophotonics. 2026 Jul 26;19(7):e70326. doi: 10.1002/jbio.70326 (PMC13401759; doi:10.1002/jbio.70326)
Supplement: Supplementary file 1 — Figure S1: Results of Principal Component Analysis (PCA) of Raman spectra from OSCC tissues of different anatomical origin. Table S1: Number of tissue samples from four areas of the oral cavity, measurement loci, number of raw and mean spectra and number of patients we collected the samples from with regard to examine the potential impact of disparate tissue sample collection sites within the oral cavity. [file JBIO-19-e70326-s001.docx]

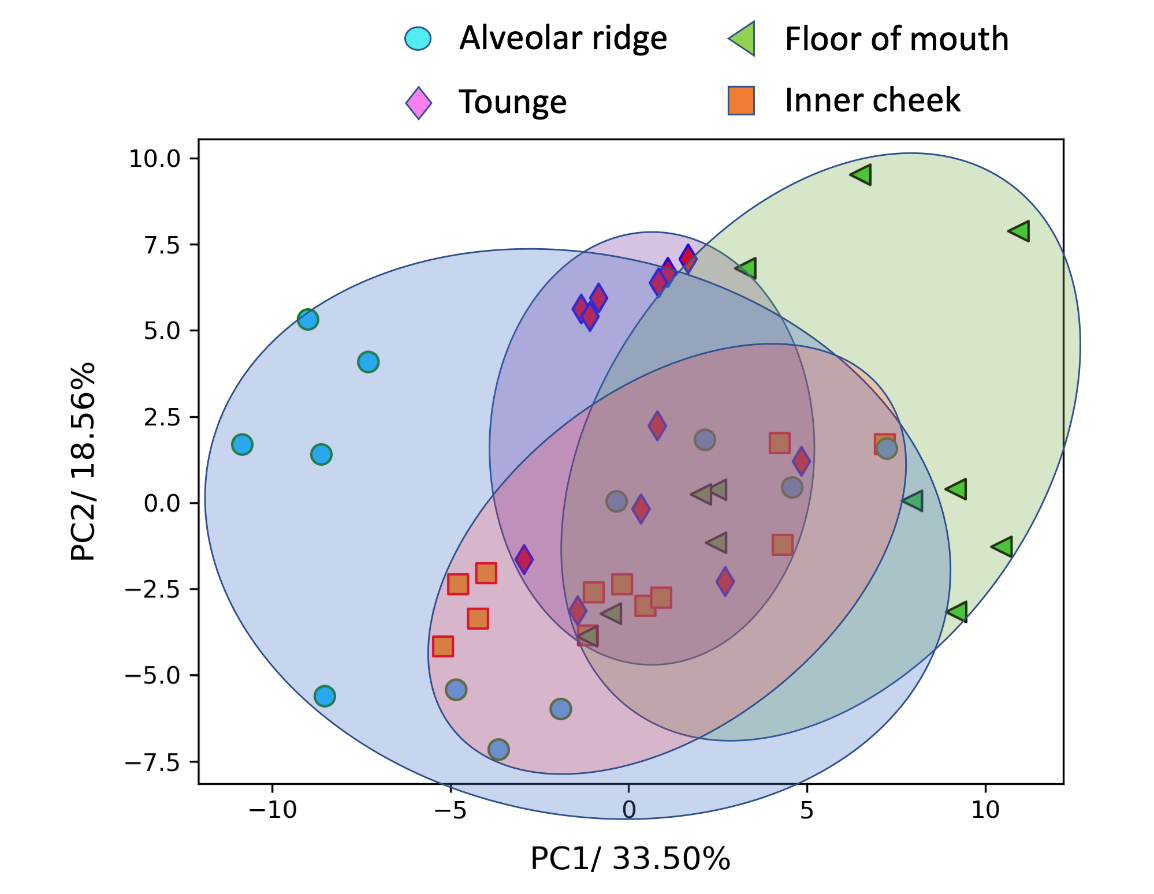


***Figure S1*** *Results of Principal Component Analysis (PCA) of Raman spectra from OSCC tissues of different anatomical origin (pink diamonds = tongue, green triangles = floor of mouth, orange squares = cheek, light blue circles = alveolar ridge). Despite clustering there is relevant overlap between the anatomical regions.*

**Table S 1** Number of tissue samples from four areas of the oral cavity, measurement loci, number of raw and mean spectra and number of patients we collected the samples from with regard to examine the potential impact of disparate tissue sample collection sites within the oral cavity.

|  | **Alveolar ridge** | **Floor of the mouth** | | **Tongue** | | **Cheek** | |
| --- | --- | --- | --- | --- | --- | --- | --- |
| tissue samples | 3 | 3 | | 3 | | 4 | |
| measurement loci | 12 | 12 | | 12 | | 12 | |
| raw spectra | 1.200 | 1.200 | | 1.200 | | 1.200 | |
| mean spectra | 24 | 24 | | 24 | | 24 | |
| number of patients | 3 | | 3 | | 3 | | 4 |
| total patients | 13 | | | | | | |
